# PSMC5 regulates microglial polarization and activation in LPS-induced cognitive deficits and motor impairments by interacting with TLR4

**DOI:** 10.1186/s12974-023-02904-9

**Published:** 2023-11-24

**Authors:** Wei Bi, Keyao Bao, Xinqi Zhou, Yihui Deng, Xiaoting Li, Jiawei Zhang, Xin Lan, Jiayi Zhao, Daxiang Lu, Yezi Xu, Yanmei Cen, Rui Cao, Mengyang Xu, Wenbin Zhong, Lihong Zhu

**Affiliations:** 1 Department of Neurology, The First Affiliated Hospital of Jinan University, No. 613, West Huangpu Avenue, Guangzhou, 510630 China; 2 Clinical Neuoscience Institute, The First Affiliated Hospital of Jinan University, No. 613, West Huangpu Avenue, Guangzhou, 510630 China; 3 Department of Pathophysiology, School of Medicine, Jinan University, No. 601, West Huangpu Avenue, Guangzhou, 510632 China; 4 Central Laboratory of the First Affiliated Hospital of Jinan University, No. 613, West Huangpu Avenue, Guangzhou, 510630 China; 5 Department of Biology, Jinan University, No. 601, West Huangpu Avenue, Guangzhou, 510632 China; 6 Guangzhou Key Laboratory for Germ-free Animals and Microbiota Application, No. 601, West Huangpu Avenue, Guangzhou, 510632 China

**Keywords:** Luteolin, Proteasome 26S subunit (PSMC5), ATPase 5, TLR4, Microglia, Synapse, Neuroinflammation

## Abstract

Luteolin is a flavonoid found in high concentrations in celery and green pepper, and acts as a neuroprotectant. PSMC5 (proteasome 26S subunit, ATPase 5) protein levels were reduced after luteolin stimulation in activated microglia. We aimed to determine whether regulating PSMC5 expression could inhibit neuroinflammation, and investigate the underlying mechanisms.BV2 microglia were transfected with siRNA PSMC5 before the addition of LPS (lipopolysaccharide, 1.0 µg/ml) for 24 h in serum free DMEM. A mouse model of LPS-induced cognitive and motor impairment was established to evaluate the neuroprotective effects of shRNA PSMC5. Intracerebroventricular administration of shRNA PSMC5 was commenced 7 days prior to i.p. injection of LPS (750 μg/kg). Treatments and behavioral experiments were performed once daily for 7 consecutive days. Behavioral tests and pathological/biochemical assays were performed to evaluate LPS-induced hippocampal damage. Molecular dynamics simulation was used to confirm the interaction between PSMC5 and TLR4 (Toll-like receptor 4) in LPS-stimulated BV2 microglia. SiRNA PSMC5 inhibited BV2 microglial activation, and suppressed the release of inflammatory factors (IL-1β, COX-2, PGE_2_, TNF-α, and iNOS) upon after LPS stimulation in BV2 microglia. LPS increased IκB-α and p65 phosphorylation, which was attenuated by siRNA PSMC5. Behavioral tests and pathological/biochemical assays showed that shRNA PSMC5 attenuated LPS-induced cognitive and motor impairments, and restored synaptic ultrastructure and protein levels in mice. ShRNA PSMC5 reduced pro-inflammatory cytokine (TNF-α, IL-1β, PGE_2_, and NO) levels in the serum and brain, and relevant protein factors (iNOS and COX-2) in the brain. Furthermore, shRNA PSMC5 upregulated the anti-inflammatory mediators interleukin IL-4 and IL-10 in the serum and brain, and promoted a pro-inflammation-to-anti-inflammation phenotype shift in microglial polarization. Mechanistically, shRNA PSMC5 significantly alleviated LPS-induced TLR4 expression. The polarization of LPS-induced microglial pro-inflammation phenotype was abolished by TLR4 inhibitor and in the TLR-4^−/−^ mouse, as in shRNA PSMC5 treatment. PSMC5 interacted with TLR4 via the amino sites Glu284, Met139, Leu127, and Phe283. PSMC5 site mutations attenuated neuroinflammation and reduced pro-inflammatory factors by reducing TLR4-related effects, thereby reducing TLR4-mediated MyD88 (myeloid differentiation factor 88)-dependent activation of NF-κB. PSMC5 could be an important therapeutic target for treatment of neurodegenerative diseases involving neuroinflammation-associated cognitive deficits and motor impairments induced by microglial activation.

**Graphical Abstract:**

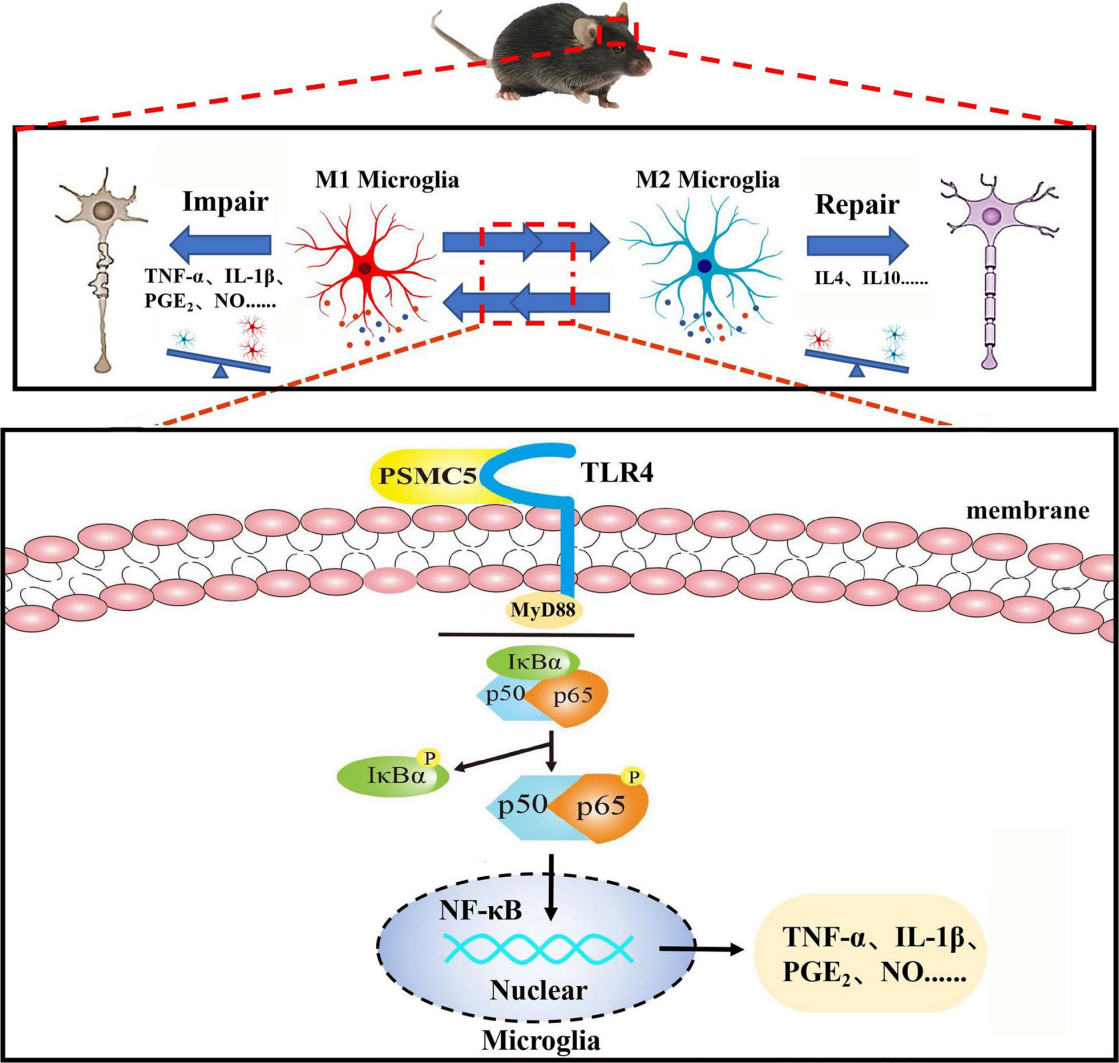

## Background

Microglia are resident innate immune cells of the central nervous system (CNS) and play an important role in host defense and tissue homeostasis in the brain [1]. Activated microglia secrete a variety of proinflammatory and neurotoxic factors that can directly affect cognition and memory [2, 3]. Inhibition of microglial activation and the resulting neuroinflammation could reduce brain damage and cognitive deficits [4].

LPS, a specific ligand for TLR4, primarily activates the MyD88-dependent and independent pathways, which involve recognition of the lipid A-region of LPS by TLR4 [5]. It can also reduce neuronal apoptosis through a mechanism involving the TLR4/MyD88/NF-κB signaling pathway in microglia [6]. In response to LPS, microglia become hyper-activated, resulting in the production of cytotoxic factors such as NO, TNF-α, and PGE_2_ [7]. Natural compounds targeting TLR4 may therefore serve as important pharmacophores for the development of potent drugs for the treatment of neurological disorders [8].

Microglial activation in the central nervous system is heterogeneous, and mainly results in formation of the contrasting M1 and M2 phenotypes [9]. The distinct functions of M1 and M2 microglia have been intensively studied. M1-activated microglia are pro-inflammatory and may contribute to the development of several CNS disorders. While the M2-activated microglia are anti-inflammatory and could promote tissue reconstruction [10]. The absence of TLR4 induces microglial polarization toward the M2 phenotype, promotes microglial migration, and therefore alleviates neuroinflammation, which indicates potential neuroprotective effects [11]. Inhibition of TLR4 expression for regulation of microglial polarization from the M1 to the M2 phenotype could prove valuable in the development of therapeutic and preventive strategies against neurodegenerative diseases [3]. Luteolin, a flavonoid found in high concentrations in celery and green pepper, could reduce the production of proinflammatory mediators in LPS-stimulated macrophages, fibroblasts, and intestinal epithelial cells [12]. We previously found that luteolin is an effective anti-inflammatory agent and may function as a neuroprotectant by inhibiting the production of pro-inflammatory factors by inhibition of NF-κB in LPS-induced BV2 microglia [13]. When hippocampal neurons were co-cultured with LPS-stimulated BV2 microglia, luteolin treatment increased neuronal viability and reduced the number of apoptotic neurons [14].

We used 2-dimensional gel electrophoresis (2-DE) based mass spectrometry (MS) to identify proteins affected by luteolin in activated microglia, and found that PSMC5 was inhibited. PSMC5 is a 19S regulatory component that can recognize ubiquitin-labeled proteins, converting them into a form suitable for degradation by the 20S complex. PSMC5 is directly involved in regulating mammalian transcription by association with transcriptionally active promotors and recruitment of coactivators [15]. However, there is increasing evidence that the proteasome also plays a role in transcription through mechanisms that do not involve proteolysis [16–18]. We used computer docking technology to determine that PSMC5 is closely related to TLR4, the mechanism by which PSMC5 promotes inflammation in vivo and in vitro remains unclear.

In this study, by using 2-dimensional gel electrophoresis (2-DE) based mass spectrometry (MS), we uncovered PSMC5 expression is reduced by luteolin and a potential target for neuroinflammation protection. Mechanically, we demonstrated PSMC5 interacts with TLR4 directly to affect TLR4-mediated MyD88-dependent signaling pathway in vivo and in vitrol. We used LPS to induce neuroinflammation, and VIPER (a specific TLR4 inhibitor), and TLR4^−/−^ mice to elucidate the mechanism of action and potential targets of shRNA PSMC5-mediated effects on cognitive and motor impairments. Subsequently, we studied the effects of shRNA PSMC5 on LPS-induced animal behaviors, microglial morphology, inflammatory factors release, protein expression, and activation of inflammatory pathways. We also aimed to demonstrate the role of PSMC5 and TLR4 in LPS-stimulated BV2 neuroinflammation by using molecular dynamics simulations to confirm the interaction between PSMC5 and TLR4, and identify the binding sites of their interaction. The interaction between PSMC5 and TLR4 was confirmed by immunofluorescence confocal and immunoprecipitation assay. GST-pull down verifies the results of the computer molecular docking and confirms the four major binding sites in the PSMC5.

## Materials and methods

### Cell culture

BV2-immortalized murine microglial cells were obtained from the Cell Culture Center of the Chinese Academy of Medical Sciences (China), and cultured in DMEM in a humidified atmosphere of 5% CO_2_ at 37 °C. The BV2 microglia were transfected with siRNA PSMC5 for 24 h, and were then treated with LPS (1.0 µg/ml) for 24 h in serum-free DMEM.

### SiRNA PSMC5

The PSMC5 siRNA and its negative control sequence were synthesized by Shanghai Gemar Pharmaceutical Technology Co., Ltd. The site was targeted at 56. It was listed in Table 1.

**Table 1:** siRNA PSMC5 targeting sequence used

mus	siRNA PSMC5
56	sense: 5ʹ-GCAGUGGACUCCGUCAAUATT-3ʹ
	antisense: 5ʹ-UAUUGACGGAGUCCACUGCTT-3ʹ

### Animals

11- to 12-week-old (22–28 g) male C57BL/6J mice (from Guangdong Medical Laboratory Animal Center), and TLR4^−/−^ knockout mice (from Model Animal Research Center of Nanjing University) were handled in accordance with the guidelines of the Animal Ethics Committee of Jinan University. All mice were housed in a room maintained on a 12/12-h light/dark cycle. The room temperature was automatically maintained at 21–25 °C with a relative humidity of 45–65%. Chow and water were provided ad libitum.

We determined whether shRNA PSMC5 protected against LPS-induced cognitive and motor impairments by inhibiting TLR4, and compared the neuroprotective effects of shRNA PSMC5 with those of VIPER, a specific TLR4 inhibitor. Eight groups of animals were used for this experimental protocol: (1) Control group; (2) Saline group; (3) shRNA PSMC5 group, LPS group; (4) VIPER group; (5) LPS group; (6) shRNA PSMC5 + LPS group; (7) VIPER + LPS group; and (8) shRNA PSMC5 + VIPER + LPS group. Mice in the VIPER + LPS group were treated with VIPER (dissolved by normal saline, 100 μg/kg, i.p.) 2 h before LPS injection (Fig. 4). Intracerebroventricular (i.c.v.) shRNA PSMC5 (lentiviruses encoding mouse shRNA PSMC5; constructed and produced by Obio Technology, Shanghai; 1 × 10^8^ TU/mL) administration was performed using a microsyringe with the stereotaxic coordinates − 0.26 cm dorsal, − 0.15 cm lateral, and − 0.02 cm anterior from bregma [19]. A dose of LPS (750 μg/kg) was injected into mice daily for 7 days.

To determine the role of TLR4 in shRNA PSMC5-mediated attenuation of cognitive impairment following neuroinflammation, animals were divided into: (1) Wild type (WT) control group; (2) WT LPS group; (3) TLR4^−/−^control group; and (4) TLR4^−/−^ LPS group. After training, testing was performed every day (day 0 to day 7).

### Two-dimensional gel electrophoresis (2-DGE) and matrix-assisted laser desorption/ionization time-of-flight mass spectrometry (MALDI–TOF-MS)

Treated cells were collected and lysed, the supernatant was subjected to 2-DGE using an Amersham Biosciences IPGphor IEF System and Hoefer SE 600 (GE healthcare, Uppsala, Sweden) electrophoresis unit (13 cm), according to manufacturer’s instructions. After 2-DGE, the gels were subjected to silver nitrate staining and scanned with an Image Scanner (GE Healthcare, Uppsala, Sweden).

Only protein spots that were consistently different in at least three independent experiments (over two-fold up- or down-regulation) were considered significant for analysis by MALDI–TOF-MS. Molecular mass analysis of the tryptic peptides was performed with an ABI 4800 plus MALDI–TOF–TOF mass spectrometer (Applied Biosystems, Foster City, CA). Spectra were interpreted and processed using the Global Protein Server Workstation (V3.6, Applied Biosystems) with the internal MASCOT search engine (V2.1, Matrix Science, London, UK) to search MS and MS/MS data. MASCOT protein scores (based on combined MS and MS/MS spectra) of > 65 were considered statistically significant (*p* ≤ 0.05). The individual MS/MS spectrum with the best ion score (based on MS/MS spectra) that was statistically significant (*p* ≤ 0.05) was also accepted.

### Real-time quantitative polymerase chain reaction (PCR) analysis

Total RNA was isolated with TRIzol reagent (Invitrogen, Carlsbad, CA) according to the instructions provided by the manufacturer. Complementary DNA was synthesized from 1 μg of total RNA with EvoScript Universal cDNA Master (Roche). The resulting cDNA was diluted and used for real-time reverse transcription PCR using a BIO-RAD PCR system. The primer sequences for the genes are listed in Table 2.

**Table 2:** Primer sequences used

IL-1β	5ʹ-CTTCCTTGTGCAAGTGTCTG-3ʹ
	5ʹ-CAGGTCATTCTCATCACTGTC-3ʹ
COX-2	5ʹ-GTGCTGGAAAAGGTTCTTCTACG-3ʹ
	5ʹ-GTGAACCCAGGTCCTCGCTTA-3ʹ
TNF-α	5ʹ-CCACCACGCTCTTCTGTCTAC-3ʹ
	5ʹ-ATCTGAGTGTGGGGTCTGG-3ʹ
GAPDH	5ʹ-TCACCACCATGGAGAAGGC-3ʹ
	5ʹ-GCTAAGCAGTTGGTGGTGCA-3ʹ

### Behavioral tests

**Fig. 1:**
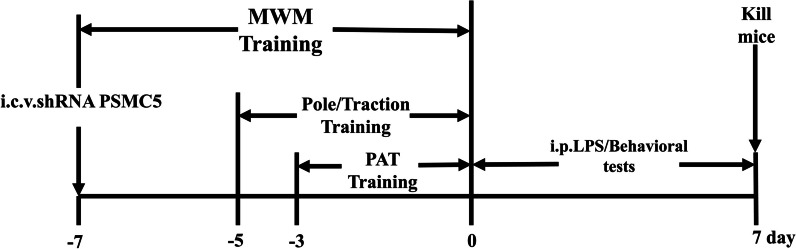
Schematic representation of experimental procedure. I.c.v. shRNA PSMC5 injection was administrated 7 days before LPS injection. Behavioral training tests were delivered to mice at days -7, -5, and -3 respectively. At day 0, behavioral tests were performed

The experimental flow chart is as Fig. 1.

### Morris water maze (MWM) test

The MWM test is widely used to assess spatial learning and memory in rodents [20]. A circular pool (height: 35 cm, diameter: 120 cm) was filled with water rendered opaque with whole milk and maintained at 23 ± 2 °C. An escape platform (height: 14 cm, diameter: 4–5 cm) was submerged in the pool 1 cm below the surface of the water in a specific position. Mice were released into the water facing the pool wall from one of four separate quadrants and were allowed to use visual tips around the pool to find the hidden platforms within 60 s. If a mouse failed to find the platform within 60 s, it was guided to the platform and allowed to stay there for 10 s. The escape latency and swimming pattern of each mouse were recorded. To assess memory consolidation, the probe trial was carried out on the 7th day. The platform was removed from the pool, and the mice were then placed into the water. The time spent in the target quadrant and the number of target-crossings were recorded.

### Passive-avoidance test

The passive-avoidance test (PAT) is a common method for evaluating memory in mice [21]. The PAT, using a “step-through” apparatus (Cheng Du Technology & Market Co, LTD.) was divided by a retractable door into two compartments: a bright compartment and a dark compartment. When the mice entered the dark compartment, they immediately received an electric shock (39 V, 3-s duration). The latency to enter the dark compartment and the number of electrical shocks (error times) within 5 min were recorded. Mice were placed in the illuminated compartment facing away from the dark compartment during the training trials for the first 3 days. A retention test was conducted again 24 h later for 7 days.

### Pole climbing test and traction test

Mouse motor behavior is evaluated using the pole test and traction test [22]. For the pole test, we conducted 5 days of training on a rough-surfaced pole (1 cm diameter and 60 cm height). After training, the pole tests were performed 7 days after LPS injection. The time taken for mice to climb down was evaluated (reaching the first half and second half of the pole and total length of the pole). The following standards were used for scoring: crossing the three parts within 3 s was scored as 3 points, within 6 s was scored as 2 points, and more than 6 s was scored as 1 point. Results were expressed as total scores.

The traction test evaluates the muscle strength of mice [23]. For this test, mice were trained to hang from a horizontal wire by their forepaws and observed for 30s for 5 days before LPS injection. If the mouse used both hind paws to catch the wire, they scored 3 points. If they used only one hind paw, they scored 2 points. If they used their forepaws, they scored 1 point, and if they dropped from the wire, they scored 0 points.

### Transmission electron microscopy (TEM)

Brain tissues were extracted in cold 4% paraformaldehyde and 2.5% glutaraldehyde solution, rinsed with 0.1 M phosphoric acid rinse solution, and fixed with 1% osmium acid fixative for 2–3 h. After fixation, brain tissues were dehydrated with ethanol and acetone. Following dehydration and embedding, brain tissues were cut using an LKB-1 ultrathin slicing machine. Finally, the samples were imaged under the transmission electron microscope (JEM, Tokyo).

### Hematoxylin and eosin (HE) staining

After 7 days of behavior tests, mice were killed and perfused using an ice-cold saline (0.9%) solution until the liver turned white. The brains were then fixed in 4% paraformaldehyde (PFA), washed 3 times with PBS for 1 h, gradient eluted using graded ethanol, and routinely processed for embedding in paraffin wax. Paraffin sections (5 μm) were then subjected to HE staining. The hippocampal tissue morphology was observed under a light microscope (Leica DMLS; Leica Microsystems Inc., Depew, New York, USA).

### Immunofluorescence staining

Immunofluorescence analyses were carried out on 10 μm-thick brain slice sequential sections prepared on a microtome (Leica CM 1850; Leica Microsystems, Seoul, Korea). The brain sections were transferred to 30% sucrose solution and permeabilized with Triton X-100 (0.3% in TBST) at room temperature for 10 min followed by three washes in 0.025% TBST, and blocked with 1% bovine serum albumin (BSA) in TBST for 1 h. After blocking, the sections were incubated at 4 °C overnight with primary antibodies against TNF-α (Abcam, Inc., Cambridge, MA, USA), chitinase 3 like protein 3 (YM-1; Abcam, Inc., Cambridge, MA, USA), or ionized calcium-binding adapter protein 1 (IBA1; Millipore Corp., Billerica, MA, USA). After washing three times with TBST, the sections were incubated with the appropriate TRIC-conjugated and DyLight 488- or 546-conjugated secondary antibodies (Invitrogen-Molecular Probes, Carlsbad, CA) at room temperature in the dark for 60 min. Nuclear staining was performed with a 4′, 6-diamidino-2- phenylindole (DAPI) staining solution for 10 min at room temperature. Finally, fluorescence images were obtained using fluorescence microscopy (Leica Microsystems, Wetzlar, Germany).

### Griess reaction and ELISA assay

Nitrite is a stable oxidative product of NO and is indirectly determined by a Griess reaction. NO production was assessed by measuring nitrite levels in the cell supernatant or serum or brain and calculated by reference to a standard curve. For the ELISA assay, serum or brain tissue was collected after treatment. IL-1 and TNF-α were measured using an ELISA kit from eBioscience (Vienna, Austria), and PGE_2_, IL-4, and IL-10 were measured using an ELISA kit from R&D Systems (Minneapolis, MN) according to manufacturers’ instructions.

### Western blot analysis

Cells or brain tissue was lysed in ice-cold radio immunoprecipitation assay (RIPA) lysis buffer (Beyotime, Beyotime Institute of Biotechnology, China) and centrifuged at 12,000*g* rpm for 20 min at 4 °C. The supernatant was collected and quantified using a BCA kit (Beyotime Institute of Biotechnology) following the manufacturer’s instructions. Cytoplasmic and nuclear p65 detection was performed using a NE-PER^®^ kit according to manufacturer’s instructions (Thermo Scientific, Rockford, IL, USA). Equal amounts of protein were separated by sodium dodecyl sulfate polyacrylamide gel electrophoresis (SDS-PAGE) and transferred onto poly vinylidene fluoride (PVDF) membranes (Millipore, Billerica, MA, USA). After blocking with 5% non-fat milk at room temperature, the membranes were incubated overnight at 4 °C with primary antibodies against PSMC5, SYP(synaptophysin), PSD95(Post; synaptic density protein 95), COX-2, iNOS, IKK-α/β, phospho-IKK-α/β (p-IKK α/β), t-IκBα, phospho-IκBα (p-IκBα), MyD88, TRIF, TLR4, GAPDH, tubulin, and lamin-B1 (Cell Signaling Technology Inc, MA, USA), and then incubated with the secondary antibody for 1 h at room temperature. The results were quantified using scanning densitometry.

### Immunoprecipitation

The BV2 microglia were washed twice with ice-cold PBS and incubated on ice for 30 min with 1 ml lysis buffer (50 mM Tris–Cl, 150 mM NaCl, 0.5 mM MgCl_2_, 10% glycerol, and 0.5% TritonX-100, pH 8) supplemented with protease inhibitor cocktail (Roche, Basel, Switzerland). Cell lysates were centrifuged for 10 min at 12,000*g*. The supernatant was preabsorbed for 1 h at 4 °C with 50 ml of protein G agarose (Thermo Fisher Scientific). The recovered supernatant was incubated with PSMC5 antibody and TLR4 antibody (all from Santa Cruz Bio-technology) at 4 °C overnight. 50 ml protein G agarose was added to the lysate-antibody mixture and incubated at 4 °C on a roller for 2 h. Agarose beads were washed 4 times with lysis buffer and boiled in 30 ml of SDS-PAGE loading buffer. Samples were resolved on 10% SDS–polyacrylamide gels and subjected to western blot analysis.

### Screening of amino acid binding sites by molecular docking and molecular simulation

Homologous models of protein TLR4 and protein PSMC5 were constructed using the software MOE 2015. 10: We used the Moe Homology Model module for homologous modeling, and Amber12 to optimize the structure of homologous models. The TLR4 and PSMC5 protein optimized homology models were used to investigate their interactions through the protein–protein docking function module in MOE 2015.10. In the functional module of MOE protein–protein interaction, the docking results were further analyzed and visualized. The sequences of protein TLR4 and protein PSMC5 were downloaded from GenBank. The template of homology modeling was identified by sequence comparison in the MOE-Search PDB module. The homology structures of TLR4 and PSMC5 proteins were constructed by software MOE 2015.10 and optimized by Molecular dynamics simulation (MD Simulation). In this study, Amber12 was used in a molecular dynamics simulation study. Adopt Amber FF03. The R1 force field uses the LEAP program to generate Molecular dynamics topology and parameter files. The TIP3P water model was used to fill the water molecules in the homologous model, which made the protein system solvable, and added ions to the system to make the system charge balance. The protein simulation system was optimized for energy, then slowly heated to 300 K, and then simulated for 300PS until the system reached thermodynamic equilibrium. Finally, the stable protein system was simulated by 10 ns long molecular dynamics, and the last 1 ns stable conformation was taken as protein–protein docking conformation.

### GST-pull down

The PSMC5 and PSMC5 mutants with GST tag were transformed into *E. coli* Rosetta (DE3) competent cells (Millipore-Sigma), which were cultured at 37 °C to an optical density at 600 nm of 0.5–1.0, followed by induction induced with 0.1 mM isopropyl b-D-1-thiogalactopyranoside for 16–18 h at 18 °C. The bacteria were collected and crude bacterial lysates were prepared by sonication in lysis buffer (50 mM Tris–Cl, 150 mM NaCl, and 1% Triton X-100,1 mM PMSF, pH8) in the presence of a protease inhibitor cocktail. Bacterial lysates were centrifuged for 20 min at 12,000*g* and the supernatants were used for fusion-protein purification with glutathione S-transferase (GST)–bind beads (Millipore-Sigma) according to the manufacturer’s protocol. HeLa cells overexpressing TLR4 were washed twice with cold PBS, lysed in lysis buffer, shaken for 30 min on ice. The lysate was cleared by 10 min of centrifugation at 12,000*g* rpm in a microcentrifuge. For pull-down, 10 mg GST or GST- PSMC5 and 30 ml GST-bind beads were incubated for 30 min on ice followed by washing 3 times with PBS. Cell lysates were then added and incubated at 4 °C overnight. The beads were washed 3 times with lysis buffer, resuspended in 23 SDS-PAGE loading buffer at 98 °C for 5 min, and resolved on 10% SDS–polyacrylamide gels for western blotting.

### Statistical analysis

Data were analyzed using SPSS 19.0 (SPSS Inc., Chicago, IL, USA) and presented as the mean ± standard error of the mean (S.E.M.). Comparisons between two groups were made using Student’s *t-*test. Comparisons among multiple groups were made using one-way ANOVA followed by Bonferroni post hoc pairwise comparisons. Repeated-measures ANOVA with Bonferroni post hoc test was used to analyze latencies in the MWM. Differences were deemed statistically significant if *P* < 0.05.

## Results

### 2-DE maps and protein identification by MALDI–TOF-MS

**Fig. 2:**
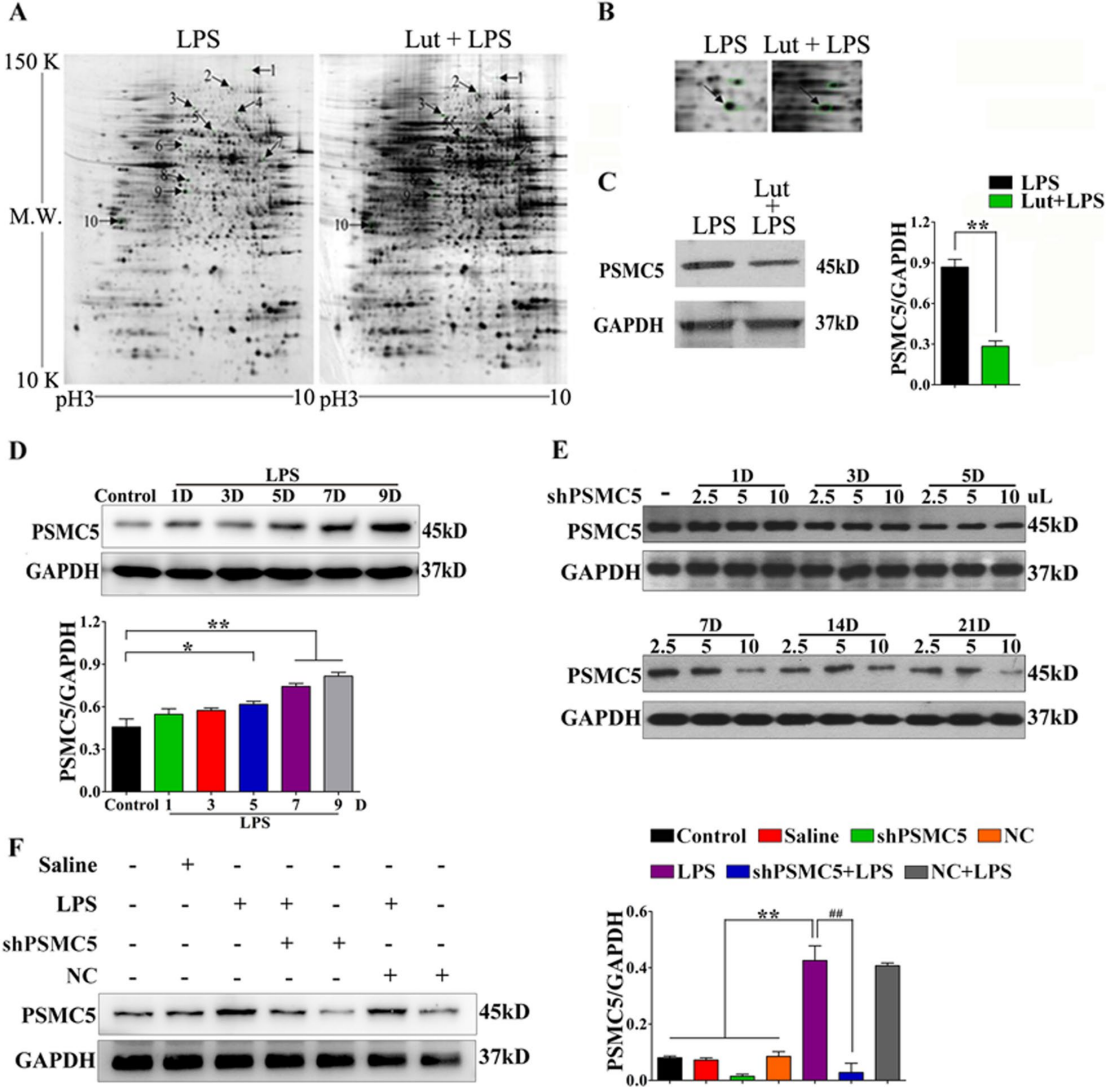
PSMC5 expression in microglia upon after luteolin treatment. **A**, **B** Protein spots identified using 2-DE gels between LPS and luteolin (Lut) treatment groups in BV2 cells, and differences in spot intensity shown by MALDI–TOF-MS to identify PSMC5. **C** Lut treatment downregulated PSMC5 protein levels in BV2 cells. *p < 0.05 and **p < 0.01 versus the LPS group. **D** PSMC5 protein levels in mouse brain at different time points in mice treated with LPS. *p < 0.05 and **p < 0.01 versus the control group. **E ** PSMC5 protein levels in the mouse brain after i.c.v. shRNA PSMC5 injection at different times and volumes. **F** PSMC5 protein levels in the mouse brain after i.c.v. shRNA PSMC5 injection. *n* = 3–4, data are expressed as means ± SEM, *p < 0.05 and **p < 0.01 versus the control, saline, shRNA PSMC5, and NC groups; ^#^*P* < 0.05, ^##^*P* < 0.01 versus the shRNA PSMC5 + LPS group

To identify the specific proteins involved in luteolin-mediated neuroprotection, we first performed proteomics analysis in BV2 cells upon after luteolin treatment. 2-DE was carried out on all the protein samples and was repeated at least thrice for each treatment. After silver nitrate staining, about 1400 spots were detected. Around 95% of the spots were matched on paired gels, and a twofold or higher difference in intensity of matched spots was considered significant. Ten protein spots were significantly different in spot intensity in all samples, and nine of them were successfully identified by MALDI–TOF-MS (Fig. 2A). Amount them, PSMC5 was significantly downregulated upon luteolin treatment (Fig. 2A, B); this reduction was confirmed by western blot analysis (Fig. 2C). These results indicated that PSMC5 may be an effective target for neuroprotection.

To study the expression of endogenous PSMC5 in the brains of LPS-induced neuroinflammation mice, we injected mice with LPS intraperitoneally for 1, 3, 5, 7, or 9 days. PSMC5 protein levels in the brain increased in a time-dependent manner upon LPS treatment (Fig. 2D). In order to decrease intracellular PSMC5 protein levels, we performed intracerebroventricular (i.c.v.) infection with a lentivirus carrying shRNA PSMC5 in the mice, which decreased PSMC5 protein levels in the absence (Fig. 2E) or presence of LPS stimulation (Fig. 2F) in the brain tissues.

### SiRNA PSMC5 suppressed LPS-induced neuroinflammation in BV2 cells

**Fig. 3:**
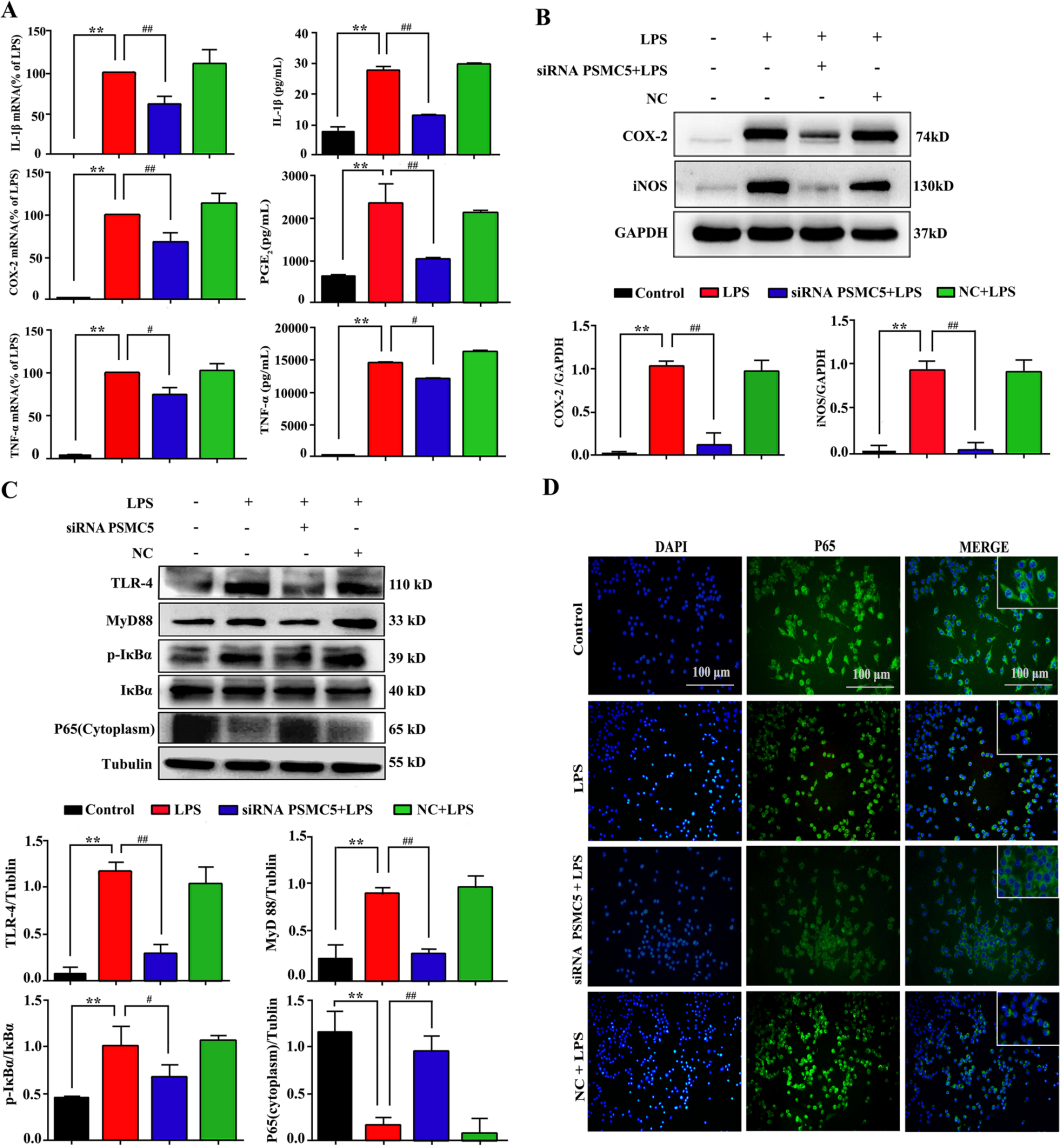
ShRNA PSMC5 suppressed neuroinflammation in BV2 cells. **A** The effect of shRNA PSMC5 on the pro-inflammatory cytokines IL-1α, COX-2, PGE_2_, and TNF-α in LPS-stimulated BV2 microglia. Q-PCR analysis of IL-1β, COX-2, and TNF-α mRNA expression. ELISA measuring the production of IL-1β, PGE_2_, and TNF-α. **B** Effect of shRNA PSMC5 on iNOS and COX-2 levels in BV2 microglia after LPS-induced neuroinflammation. **C** Signaling components of MyD88-dependent signaling pathway analyzed in BV2 using western blot with the indicated antibodies. **D** Cellular distribution of NF-κB p65 subunit (Green). Hoechst 33,258 (blue) was used for visualizing nuclei. Scale bar = 100 μm

Microglial activation and cytokines are essential for LPS-induced neuroinflammation. To identify the inflammatory factors released at the mRNA and protein levels after siRNA PSMC5 transfection, we examined IL-1β, COX-2, PGE_2_, TNF-α, and iNOS expression. As shown in Fig. 3A, IL-1β, PGE_2_, and TNF-α levels increased after LPS; siRNA PSMC5 significantly reduced their expression (*P* < 0.01). In addition, the increase in COX-2 and iNOS protein levels observed with LPS stimulation was reversed by siRNA PSMC5 expression (*P* < 0.01, Fig. 3B).

To determine whether siRNA PSMC5 affects TLR4-MyD88 signaling in BV2, we studied the activation of the TLR4-MyD88 signaling pathway. TLR4 and MyD88 protein levels and IκB-α phosphorylation were significantly enhanced, and cytoplasmic p65 was significantly decreased after LPS treatment. SiRNA PSMC5 treatment attenuated the expression of TLR4 and MyD88 and IκB-αphosphorylation, and promoted nuclear translocation of the p65 subunit (Fig. 3C and D), indicating TLR-MyD88 signaling is involved in PSMC5 mediated neuroinflammation upon LPS stimulation.

### ShRNA PSMC5 could inhibit LPS-induced cognitive and motor dysfunction

To determine whether shRNA PSMC5 expression alters cognitive and motor behavior, the MWM, PAT, pole, and traction tests were performed to assess spatial learning and memory in mice. We also administered a TLR4 inhibitor (VIPER) in LPS-induced mice to elucidate the effect of shRNA PSMC5 on cognitive and motor function.

**Fig. 4:**
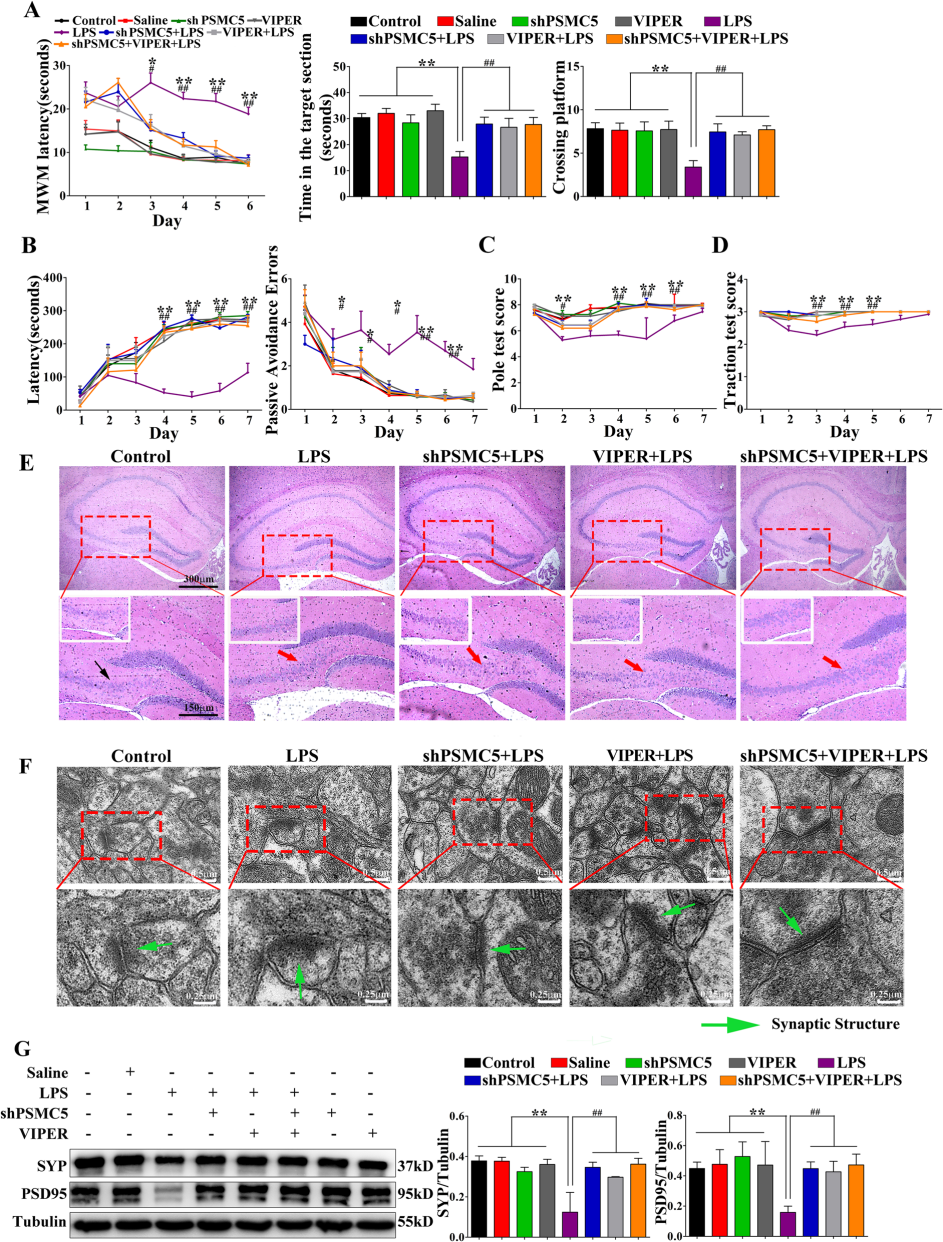
ShRNA PSMC5 alleviated cognitive impairment and motor disjunction, and improved synaptic structure in LPS-induced mice. **A** Results of the MWM test for shRNA PSMC5-treated mice, *n = *15. **B** Results of the PAT test for shRNA PSMC5-treated mice, *n = *15. **C**, **D** Motor coordination scores for shRNA PSMC5-treated mice. **C** Pole test score, *n = *15. **D** Traction test score, *n = *15. Data are presented as mean ± SEM. *P < 0.05, **P < 0.01 compared to the control, saline, shRNA PSMC5, and VIPER groups; ^#^*P* < 0.05, ^##^*P* < 0.01 compared to the shRNA PSMC5 + LPS, VIPER + LPS, and shRNA PSMC5 + VIPER + LPS groups, analyzed by one-way ANOVA. Error bars indicate SEM. **E** Hematoxylin and eosin staining shows effect of shRNA PSMC5 on hippocampus neurons in LPS-induced mice. Representative photomicrographs of the hippocampus CA3 and CA4 area showing the histological changes of each group. *n = *5 mice/group, *n = *30 felds/group. **F** Transmission electron microscopy staining shows effects of shRNA PSMC5 treatment on synapses in the hippocampus. The green arrow shows the synaptic structure. *n = *5 mice/group, *n = *30 felds/group. **G** Effect of shRNA PSMC5 treatment on SYP and PSD95 levels in the mice brain. *n = *3–4 mice/group

Mice what received LPS treatment exhibited an increased latency to reach the hidden platform in the acquisition phase of the MWM, suggesting that LPS induced memory deficits. Mice that received i.c.v injections of shRNA PSMC5 displayed significantly lower escape latency after LPS treatment. In the spatial probe test, the mean incidences of crossing the removed platform and time in target section were higher in shRNA PSMC5-pretreated mice than in untreated controls after LPS treatment (Fig. 4A). In the PAT, shRNA PSMC5-pretreated mice showed significant amelioration of hippocampal cognitive function—longer latency and less errors—than did LPS mice (Fig. 4B). As shown in Fig. 3C and D, test scores in shRNA PSMC5-pretreated mice were significantly higher than those in LPS treated mice, indicating that shRNA PSMC5 treatment improved locomotive performance. ShRNA PSMC5 and VIPER had similar effects on cognitive impairment and motor dysfunction.

### ShRNA PSMC5 protects synaptic ultrastructure and synaptic protein expression

To observe the hippocampal neurons, we performed HE staining on brain tissues from each group of mice (Fig. 4E).

The difference in hippocampal neurons indicated that reinstatement of synaptic function was accompanied by a reversal of synaptic loss. We then observed the ultrastructure of the synapses and quantified the synaptic protein levels in the hippocampus. To observe the ultrastructure of the synapses in the hippocampus, we conducted electron microscopy. After LPS injection, the density of the synaptic connections and the number of synapses were decreased. These alterations were reversed by shRNA PSMC5 administration, and the mice exhibited healthier synaptic ultrastructure, including more synapses and greater synaptic connection density (Fig. 4F).

Western blotting for synaptic proteins revealed that the presynaptic protein synaptophysin, the postsynaptic protein PSD95, and the scaffold protein spinophilin, which were markedly reduced in LPS mice, were significantly upregulated in the hippocampal homogenates of shRNA PSMC5 mice (Fig. 4G).

### ShRNA PSMC5 regulated microglial polarization and suppressed neuroinflammation in LPS-induced mice

**Fig. 5:**
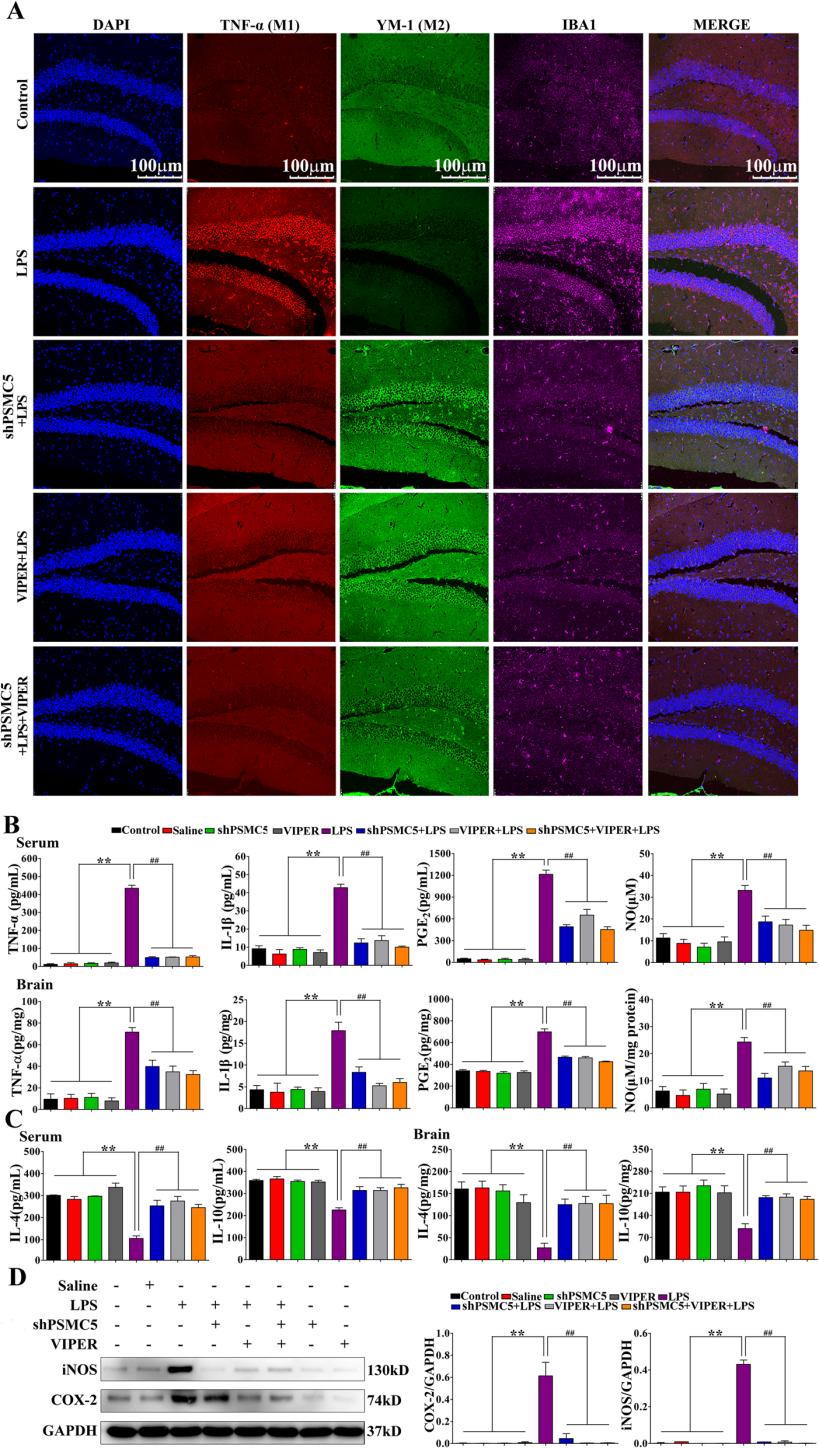
ShRNA PSMC5 treatment shifted microglial polarization from pro-inflammatory phenotypes toward to anti-inflammatory phenotypes after LPS-induced neuroinflammation. **A** ShRNA PSMC5 decreased the number of TNF-α-positive microglia (M1) and increased the number of YM-1-positive microglia (M2) in the hippocampus. Representative images of triple-staining immunofluorescence for TNF-α (red), YM-1 (green), and IBA-1 (purple) with DAPI nuclear counterstain in the hippocampus. **B** ShRNA PSMC5 treatment decreased LPS-induced pro-inflammatory cytokines in the serum and brain. Pro-inflammatory cytokines were detected by ELISA and Griess assay. **C** ShRNA PSMC5 treatment increased LPS-induced anti-inflammatory cytokine production in the serum and brain. Anti-inflammatory cytokines were detected by ELISA. **D** Effect of shRNA PSMC5 treatment on iNOS and COX-2 levels in the brain after LPS-induced neuroinflammation. ShRNA PSMC5 treatment alleviated the expression of iNOS and COX-2 protein. *n = *3–4 mice/group.Data are presented as mean ± SEM. *P < 0.05, **P < 0.01 compared to the control, saline, shRNA PSMC5, and VIPER groups; ^#^*P* < 0.05, ^##^*P* < 0.01 compared to the shRNA PSMC5 + LPS, VIPER + LPS, and shRNA PSMC5 + VIPER + LPS groups, analyzed by one-way ANOVA. Error bars indicate SEM

LPS increased the number of both IBA1 and TNF-α-positive microglia in the hippocampus. This number decreased after shRNA PSMC5 injection (Fig. 5A). Microglial activation and cytokines are essential for LPS-induced neuroinflammation. We therefore monitored the levels of specific proinflammatory cytokines (TNF-α, IL-1β, PGE_2_, and NO) in serum and brain homogenates, and inflammation-related protein factors (iNOS and COX-2) in the brain. ELISA and Griess assay revealed that TNF-α, IL-1β, PGE_2_, and NO levels increased after LPS i.p. injection. ShRNA PSMC5 treatment significantly reduced their expression in the serum and brain (Fig. 5B). In addition, the iNOS and COX-2 protein levels in the brain of shRNA PSMC5 mice were significantly lower than those in LPS mice (Fig. 5D).

We subsequently detected the levels of anti-inflammatory markers (IL-4 and IL-10) in serum and brain homogenates to assess anti-inflammatory effects. As shown in Fig. 5C, shRNA PSMC5 treatment significantly increased IL-4 and IL-10 levels in the serum and brain after LPS injection.

### ShRNA PSMC5 could inhibit LPS-induced TLR4-MyD88-dependent signaling pathway activation

To determine whether shRNA PSMC5 treatment affects NF-κB signaling in our model, we examined the activation of this signaling pathway.

**Fig. 6:**
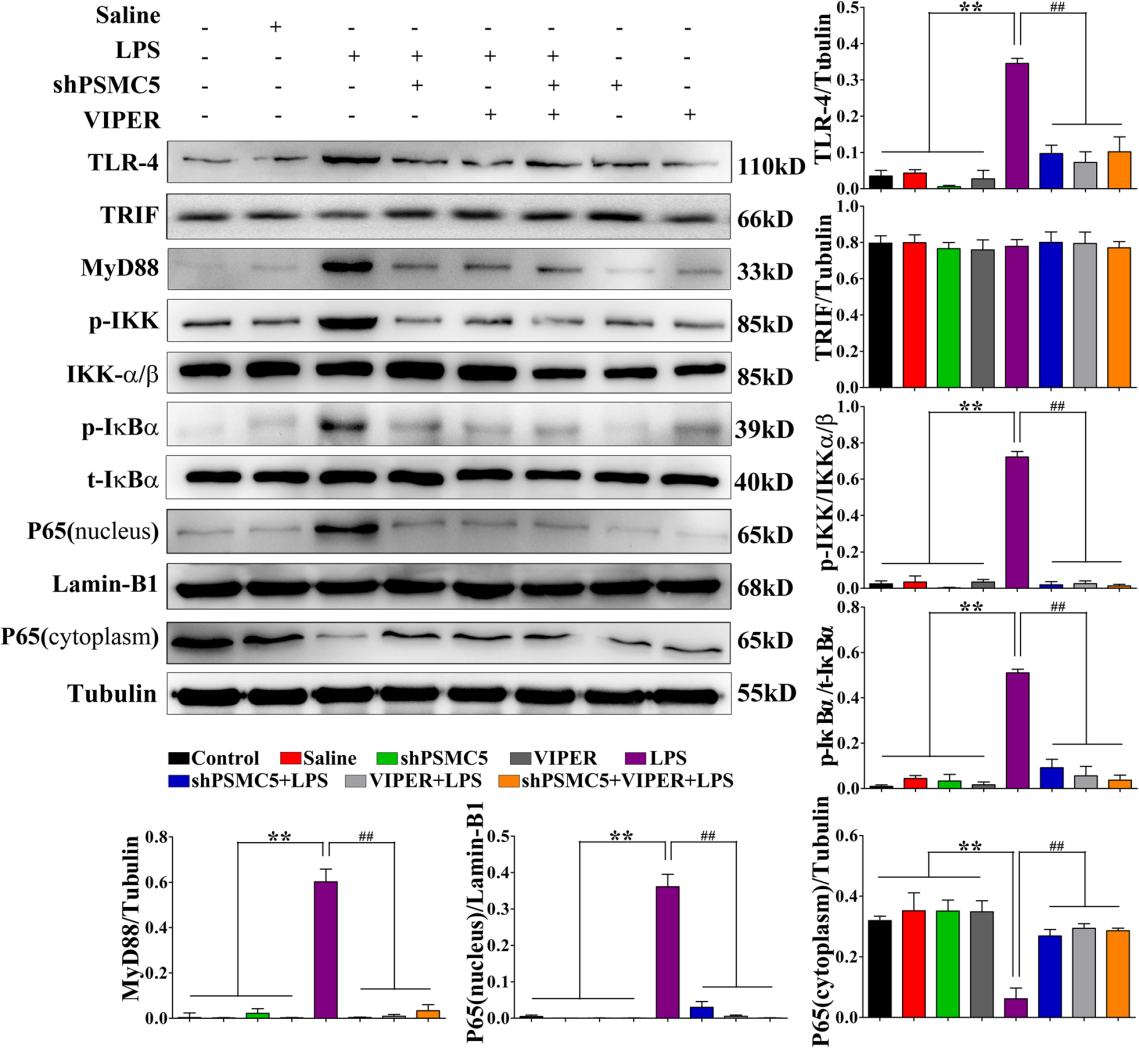
ShRNA PSMC5 inhibited MyD88-dependent signaling pathway activation and suppressed transition of microglial polarization from M2 to M1 phenotype under neuroinflammatory conditions. Protein expression of signaling components of the MyD88-dependent pathway were analyzed using the indicated antibodies by western blot. *n = *3–4 mice/group.Data are presented as mean ± SEM. *P < 0.05, **P < 0.01 compared to the control, saline, shRNA PSMC5, and VIPER groups; ^#^*P* < 0.05, ^##^*P* < 0.01 compared to the shRNA PSMC5 + LPS, VIPER + LPS, and shRNA PSMC5 + VIPER + LPS groups, analyzed by one-way ANOVA. Error bars indicate SEM

TLR-4 and MyD88 expression and IκBα and IκB kinase (IKK) phosphorylation in the LPS groups were significantly higher than those in the control groups. This increase induced the translocation of the NF-κB p65 subunit into the nucleus (Fig. 6). There were no significant differences in TRIF protein expression between different groups. Furthermore, shRNA PSMC5 treatment reduced TLR-4 and MyD88 expression, attenuated the phosphorylation of IKK and IκB-α, and inhibited nuclear translocation of the p65 subunit, which was similar to the effects of VIPER.

### Cognitive deficits, motor impairment, and inflammation in TLR4 knock out mice (TLR4^−/−^)

**Fig. 7:**
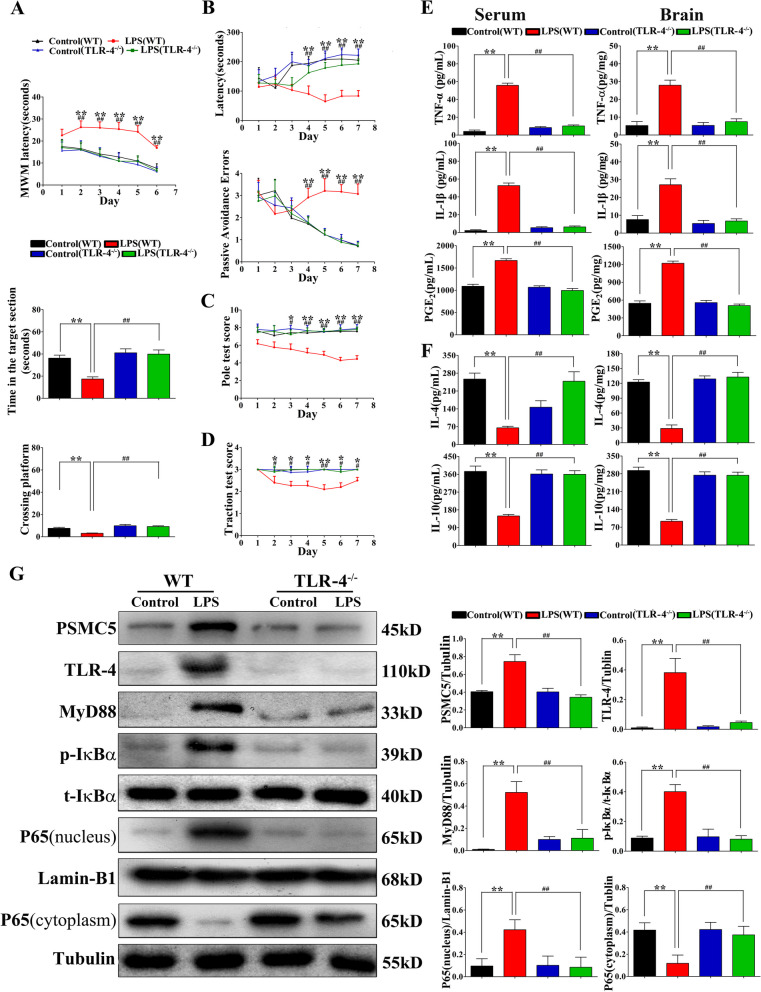
TLR4 knockout in mice protected against LPS-induced neuroinflammation and cognitive and motor impairments. **A**–**D** Morris water maze (MWM), passive avoidance test (PAT), pole test, and traction test were performed to test the memory ability and motor coordination in mice that received the indicated treatments. *n = *15. **E** The expression of the pro-inflammatory cytokines TNF-α, IL-1β, and PGE_2_ in mouse serum and brain were determined using ELISA kits. **F** Expression of the anti-inflammatory cytokines IL-4 and IL-10 in mouse serum and brain were studied using ELISA kits. **G** ShRNA PSMC5 targeted TLR4 to ameliorate LPS-induced neuroinflammation. The expression levels of the signaling components of the TLR4-pathway in mouse hippocampus were determined using western blot with the indicated antibodies. **E**–**G** *n = *3–4 mice/group,data are presented as mean ± SEM. *P < 0.05, **P < 0.01 compared to control group; ^#^*P* < 0.05, ^##^*P* < 0.01 compared to LPS (TLR4^−/−^) group, analyzed by one-way ANOVA. Error bars indicate SEM

We determined whether TLR4 deficiency affects cognitive impairment after LPS-induced neuroinflammation by injecting TLR4 knockout mice (TLR4^−/−^) with LPS and evaluating memory and motor function. TLR4^−/−^ mice reached the platform faster, and spent more time in the platform quadrant and on the platform, as compared to WT mice (Fig. 7A). In the PAT, TLR4^−/−^ mice spent less time in the dark compartment and showed fewer errors in the passive avoidance test than did WT mice (Fig. 7B). Motor coordination scores of TLR4^−/−^mice were significantly higher than those of WT mice (Fig. 7C, D). Furthermore, TLR4^−/−^mice exhibited lower levels of pro-inflammatory cytokines, such as TNF-α, IL-1β, and PGE_2_ in the serum and brain (Fig. 7E). We subsequently detected the levels of anti-inflammatory markers in serum and brain homogenates. As shown in Fig. 7F, TLR4^−/−^mice exhibited higher levels of IL-4 and IL-10.

We then assessed hippocampal TLR4, MyD88, p-IκB-α, p65, and PSMC5 protein expression in TLR4^−/−^and WT mice. WT mice showed significantly higher TLR4, MyD88, p-IκB-α, and p65 protein expression than that in TLR4^−/−^mice (Fig. 7G). PSMC5 expression was increased after LPS injection in WT mice. However, TLR4 knockout abolished LPS-induced PSMC5 upregulation (Fig. 7G). These results corresponded with those observed (behavior, pro-inflammatory cytokines, and signaling pathway activity) in VIPER-treated mice (Figs. 4–6). Thus, shRNA PSMC5-treated mice exhibited similar protective effects to those in TLR4^−/−^ mice and VIPER-treated mice.

### Molecular dynamics simulation to identify the interaction between PSMC5 and TLR4 in LPS-stimulated BV2 microglia

**Fig. 8:**
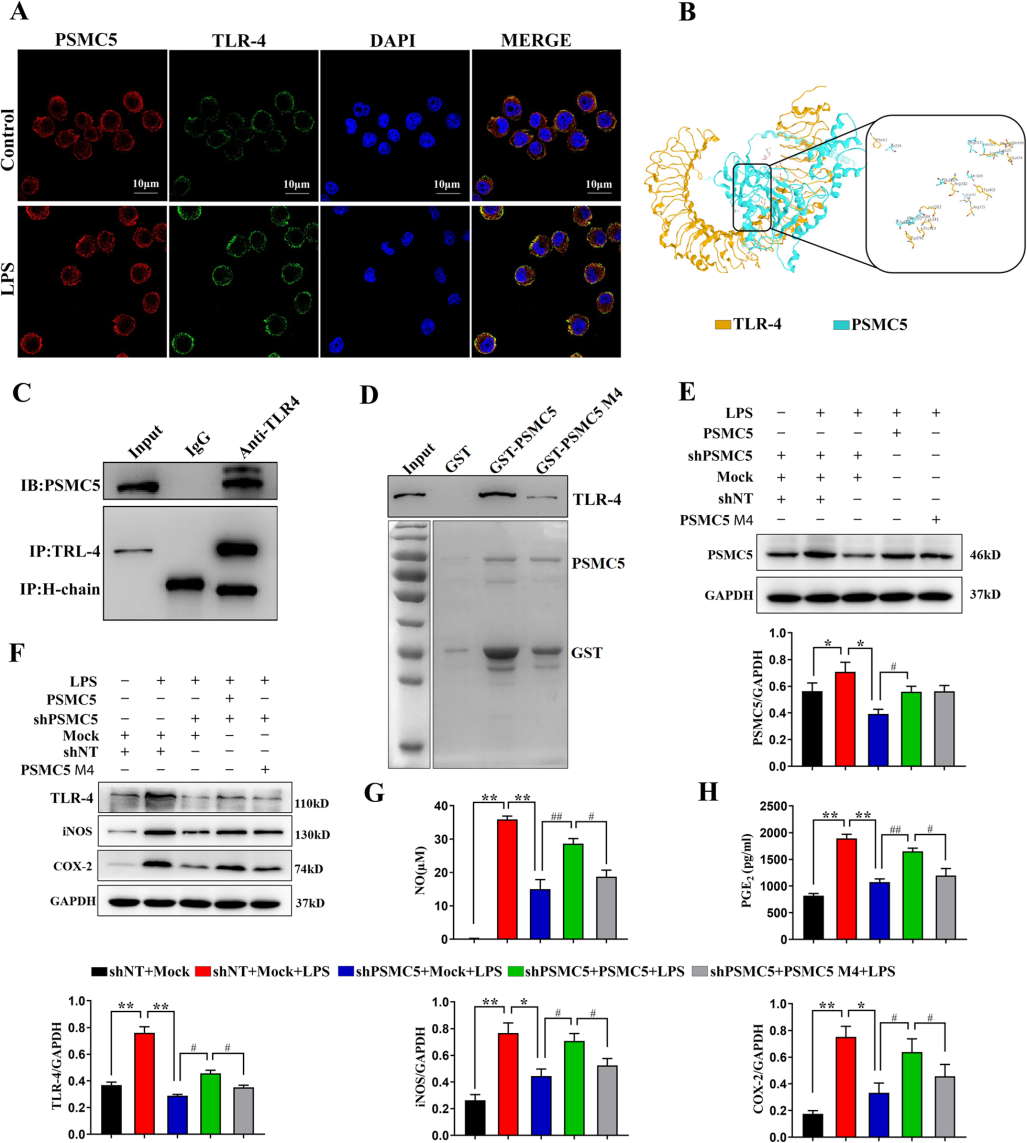
PSMC5 interacts with TLR4. **A** Colocalization of PSMC5 (red) and TLR4 (green), with DAPI nuclear counterstain in the BV2 microglia. Scale bar = 10 µm. **B** In silico modeling of PSMC5 interactions with TLR4. **C** Coimmunoprecipitation of endogenous PSMC5 and TLR4 in BV2 cells; precipitation was carried out with TLR4 antibody or IgG followed by WB analysis with antibodies against PSMC5 and TLR4. **D** GST-pull down to verify the computer molecular docking results. **E** PSMC5 expression after PSMC5 knockdown in the PSMC5 wild-type and PSMC5 mutants. **F** Western blot analysis of protein expression of the inflammatory proteins iNOS and COX-2, and MyD88-dependent signaling pathway components in the PSMC5 wild-type and mutants. **G** Results of the Griess assay showing NO content in the PSMC5 mutants. **H** ELISA analysis showing the PGE_2_ content in the PSMC5 mutants

Confocal immunofluorescence revealed the co-localization of PSMC5 and TLR4 in BV2 cells (Fig. 8A). Residues of PSMC5 (Glu284, Met139, Leu127, and Phe283) are essential for its binding to TLR4, as determined by molecular docking (Fig. 8B).

Co-immunoprecipitation confirmed their interaction (Fig. 8C). We then performed GST-pull down to verify the molecular docking results (Fig. 8D). After PSMC5 knockdown, the wild-type and mutant PSMC5 could overexpress PSMC5, and the expression levels were as expected (Fig. 8E). Interaction between PSMC5 and TLR4 is attenuated by mutations in four major sites of PSMC5. Western blot showed that, compared with the wild-type, the expression of the inflammatory proteins iNOS and COX-2 and the signaling pathway protein TLR4 was decreased in the PSMC5 mutants (Fig. 8F). The results of the Griess test showed that NO content in the PSMC5 mutants was significantly lower than that in the wild type (Fig. 8G). ELISA analysis showed that the PGE_2_ content in the PSMC5 mutants was lower than that in the wild-type (Fig. 8H). These results indicated that PSMC5 regulates inflammation by directly binding to TLR4.

## Discussion

There is increasing evidence that luteolin can regulate the activation of microglia. Preincubation of microglia with luteolin diminished neurotoxic effects, owing to its direct anti-inflammatory effects [13]. Luteolin suppresses NF-κB, STAT1, and IRF-1 signaling, thus attenuating the inflammatory response in brain microglial cells [24]. Luteolin inhibits LPS-induced IL-6 production in the brain by inhibiting the JNK signaling pathway and activating AP-1 in microglia [12]. Luteolin could also inhibit microglial inflammation by enhancing USP8 [25]. The mechanism underlying the effects of luteolin are unclear; we therefore used proteomics to elucidate this mechanism. PSMC5 was identified by 2-DE and MS techniques in activated microglia treated with luteolin. Our results provide a new potential target for insights into the mechanism of luteolin.

The 26S proteasome has intrinsic ATPase activity that could play an essential role in its proteolytic function [26]. At least 6 different species of ATPase belonging to the same family, including PSMC5, have been associated with the human 26S proteasome [27]. PSMC5 was mapped to human chromosomes 17 q23.1-q23.3 [28], and was originally identified as a suppressor (in mutant form) of a mutant allele of the transcription factor GAL4 [29]. PSMC5 was subsequently identified as a component of a multiprotein mediator of transcription factor-dependent transcription by RNA polymerase II (pol II) holoenzyme from S. cerevisiae [30]. PSMC5 interacts with various transcription factors [31], and the proteasomal PSMC5 subunit could be recruited by liganded nuclear receptors to selectively specify their own degradation [32]. PSMC5 plays a distinct proteasome-independent role in regulating transcription activation and elongation, DNA repair, and chromatin remodeling [33]. However, the mechanism by which PSMC5 inhibits inflammation in vivo and in vitro remains unclear.

LPS are widely used in experimental models of systemic bacterial infection and trigger robust inflammation by potently activating TLR4 expressed on innate immune cells [34]. LPS stimulates BV2 microglia cells to activate the TLR4-MyD88 signaling pathways and release various inflammatory factors such as IL-1β, IL-6, and TNF-α [35], leading to neuron apoptosis [36]. Inhibition of TLR4 signaling reduces the risk of neurodegenerative diseases, and up-regulation of anti-inflammatory cytokines associated with LPS hyperresponsiveness can have adverse effects in the brain [37]. As shown in Fig. 3A, the levels of IL-1β, PGE_2_, and TNF-α increased after LPS, and siRNA PSMC5 significantly reduced their expression (*P* < 0.01). In addition, the COX-2 and iNOS protein levels in the siRNA PSMC5 group were significantly lower than those in the LPS groups (*P* < 0.01, Fig. 3B). Our results also revealed that IκB-α phosphorylation in the LPS group was significantly higher than that in the siRNA PSMC5 group, which showed nuclear translocation of the p65 subunit. (Fig. 3C, D). These results indicate that siRNA PSMC5 inhibits BV2 microglial activation. The exact molecular mechanisms by which PSMC5 regulates microglia and its impact on cognitive and motor impairments merit further study.

The hippocampal neuronal system has a higher microglial proliferative capability after LPS-induced inflammation compared with other brain regions [38]. LPS treatment leads to cognitive impairment in mice, as shown by the Morris water maze and passive avoidance tests, and these effects were accompanied by microglial activation [39]. LPS injection induced a time-dependent increase in endogenous PSMC5 levels. In this study, MWM results indicated that LPS treatment prolonged the escape latency, and these effects and with the number of errors showed significant improvement at the begin of the third day after shRNA PMSC5 treatment. In addition, the time spent in the target quadrant and number of platform crossings were significantly improved after shRNA PMSC5 treatment. Furthermore, shRNA PMSC5 increased the scores on the pole climbing test. Thus, shRNA PMSC5 ameliorated the cognitive and motor impairment caused by LPS. These findings suggest a potential application for shRNA PMSC5 in patients with or at risk for cognitive and motor impairment induced by microglial activation.

Synaptic plasticity is the basis of learning and memory [40] and hippocampal synaptic function damage occurs before memory loss, Aβ deposition, and neuronal cell death [41]. Postsynaptic density protein 95 (PSD-95) and synaptophysin (SYP) are closely related to synaptic function, and PSD95 has an important role in synaptic plasticity and stability as well as peripheral nerve repair after injury [42]. As a presynaptic plasticity related protein, changes in SYP expression could indirectly affect the number, distribution, and density of synapses [43]. We found that shRNA PSMC5 improved synaptic connections in LPS-induced mice; the mice exhibited healthier synaptic ultrastructure, more synapses, and greater synaptic connection density (Fig. 4F). Western blotting revealed that shRNA PSMC5 significantly upregulated synaptic proteins (PSD-95 and SYP) in the hippocampal homogenates (Fig. 4G). Thus, shRNA PSMC5 improved synaptic ultrastructure and protein levels, which were particularly important for cognitive function.

Microglial activation and cytokines are essential in LPS-induced neuroinflammation. Under the influence of endogenous or pathological signals, microglia undergo biochemical transformations that are classified as the pro-inflammatory M1 phenotype and the alternatively activated M2 state [44]. Manipulation of microglia phenotypes from the pro-inflammatory, cytotoxic M1 to anti-inflammatory, neuroprotective M2 could be a therapeutic approach for some neurodegenerative diseases associated with neuroinflammation such as Alzheimer’s and Parkinson’s diseases, and amyotrophic lateral sclerosis [9]. TNF-α is a classic marker of activated microglia (M1) and YM-1(chitinase 3 like protein 3) is a marker of alternatively activated microglia (M2) [45]. Localization of IBA1 (ionized calcium-binding adapter protein 1) protein is restricted to microglia in vitro and in vivo, and IBA1 protein plays a role in regulating microglial function, especially in activated microglia [46]. In this study, we showed that LPS increased the number of IBA1 and TNF-α-positive microglia in the hippocampus, and this number decreased after shRNA PSMC5 injection (Fig. 5A). ShRNA PSMC5 suppressed cognitive and motor impairments after neuroinflammation by significantly decreasing M1 phenotype-associated pro-inflammatory cytokines (TNF-α, IL-1β, PGE_2_, and NO), and increasing anti-inflammatory cytokine (IL-4 and IL-10) expression (Fig. 5B, C). In addition, the iNOS and COX-2 protein levels in the brain of shRNA PSMC5 mice were significantly lower than those in LPS mice (Fig. 5D). These results indicate that shRNA PSMC5 in the brain promotes the polarization of microglia to the M2 phenotype and inhibits microglial activation. The change of polarization state plays an important role in the regulation of inflammatory factors.

TLR4 can recognize conserved motifs in various pathogens and mediate defense responses [47]. Triggering the TLR4 pathway often leads to activation of NF-κB and subsequent regulation of immune and inflammatory genes [48]. After activation, TLR4 interacts with many cytoplasmic adaptor proteins, including MyD88, MyD88 like adaptor protein/TIR related adaptor protein (MAL/TIRAP), toll receptor associated interferon activator (TRIF), and toll receptor related molecules [49]. This association results in the recruitment and activation of IRAK1 (Interleukin 1 receptor associated kinase 1) and IRAK4 (Interleukin 1 receptor associated kinase 4) to form complexes with TRAF6 (TNF receptor associated factor 6) to activate TAK1 (Human Transforming growth factor kinase 1) and IKK(inhibitor of kappa B kinase). IKK activation can lead to the degradation of IκB; IκB promotes the retention of NF-κB in the cytoplasm in an inactive state [50]. Our results revealed that shRNA PSMC5 treatment decreased TLR4 and MyD88 levels, attenuated the phosphorylation of IKK and IκB-α, and inhibited nuclear translocation of the p65 subunit, similar to the effects of VIPER (Fig. 6). These data indicate the involvement of the MyD88-dependent signaling pathway in activating inflammation, resulting in the activation of microglia and subsequent cognitive impairment. Notably, shRNA PSMC5 treatment inhibited TLR4-mediated neuroinflammation. The regulation of microglia activation can inhibit the release of TLR4 receptor-mediated transcriptional factors (TNF-α, IL-β, PGE_2_, and NO), thus protecting neurons, is an important strategy for drug development.

We used VIPER and TLR4^−/−^ mice to compare the protective effects of shRNA PSMC5. The results from VIPER administration and the KO mouse model indicated that, similar to shRNA PSMC5, blocking TLR4 attenuated cognitive and motor impairments. Moreover, NF-κB activated by LPS triggered neuroinflammation in neuronal cells by activation of microglial cells via a series of inflammatory cytokines; however, this phenomenon was suppressed in VIPER-treated mice or TLR4^−/−^ mouse brains (Fig. 7). These findings provide strong evidence that shRNA PSMC5-treated mice exhibited similar protective effect to those in TLR4^−/−^ mice and VIPER-treated mice.

Computer simulation of the binding patterns of protein molecules to identify specific binding sites between proteins provide a basis for experimental work and help understand molecular interactions [51]. The GST-pull down technique was used to confirm the predicted protein–protein interactions [52]. Co-immunoprecipitation is used to isolate and study the natural state of protein interaction complexes [53]. We identified PSMC5 binding patterns to TLR4 protein molecules by silico techniques. CO-IP (co-immunoprecipitation) and confocal immunofluorescence confirmed the interaction between PSMC5 and TLR4. We identified specific residues in PSMC5 (Glu284, Met139, Leu127, and Phe283) that function as the binding sites for TLR4 (Fig. 8B), and used GST-pull down to confirm the computer molecular docking results. Interaction between PSMC5 and TLR4 could be attenuated by the mutating four major sites in PSMC5 (Fig. 8D). We rescued wild-type and mutant PSMC5 expression in PSMC5 knockdown cells (Fig. 8E); mutant PSMC5 expression did not rescue iNOS and COX-2 expression (Fig. 8F) or NO and PGE_2_ content in PSMC5 knockdown cells (Fig. 8G, H). These data indicated a direct interaction between PSMC5 and TLR4. PSMC5 mutants may attenuate neuroinflammation and reduce pro-inflammatory factors by reducing TLR4-related effects, thereby reducing TLR4-mediated MyD88-dependent activation of NF-κB.

## Conclusion

Our results demonstrated that regulating the expression of PSMC5 may attenuate neuroinflammation and reduce pro-inflammatory factors by reducing TLR4-related effects, thereby reducing TLR4-mediated MyD88-dependent activation of NF-κB. Regulating the expression of PSMC5 could have important therapeutic implications for treating neurodegenerative diseases involving neuroinflammation-associated cognitive deficits and motor impairments induced by microglial activation.

## Data Availability

All data generated or analyzed during this study are included in this published article.

## Abbreviations

PSMC5: Proteasome 26S subunit, ATPase 5
LPS: Lipopolysaccharide
TLR4: Toll-like receptor 4
CNS: Central nervous system
2-DE: 2-Dimensional gel electrophoresis
MS: Mass spectrometry
2-DGE: Two-dimensional gel electrophoresis
MALDI–TOF-MS: Matrix-assisted laser desorption/ionization time-of-flight mass spectrometry
PCR: Real-Time Quantitative Polymerase Chain Reaction
MWM: Morris water maze
PAT: Passive-avoidance test
TEM: Transmission electron microscopy
HE: Hematoxylin and eosin
BSA: Bovine serum albumin
SDS-PAGE: Sodium dodecyl sulfate polyacrylamide gel electrophoresis
PVDF: Poly vinylidene fluoride
SYP: Synaptophysin
PSD-95: Post synaptic density protein 95
YM-1: Chitinase 3 like protein 3
IBA1: Ionized calcium-binding adapter protein 1
MyD88: Myeloid differentiation factor 88
TRIF: Toll receptor associated interferon activator
IRAK1: Interleukin 1 receptor associated kinase 1
IRAK4: Interleukin 1 receptor associated kinase 4
TRAF6: TNF receptor associated factor 6
TAK1: Human Transforming growth factor kinase 1
IKK: Inhibitor of kappa B kinase
CO-IP: Co-immunoprecipitation
